# Native α-Synuclein, 3-Nitrotyrosine Proteins, and Patterns of Nitro-α-Synuclein-Immunoreactive Inclusions in Saliva and Submandibulary Gland in Parkinson’s Disease

**DOI:** 10.3390/antiox10050715

**Published:** 2021-05-01

**Authors:** Emilio Fernández-Espejo, Fernando Rodríguez de Fonseca, Juan Suárez, Eduardo Tolosa, Dolores Vilas, Iban Aldecoa, Joan Berenguer, Fátima Damas-Hermoso

**Affiliations:** 1 Reial Acadèmia de Medicina de Catalunya, 08001 Barcelona, Spain; 2 Red Andaluza de Investigación Clínica y Traslacional en Neurología (Neuro-RECA), Laboratorio de Medicina Regenerativa, Hospital Regional Universitario, 29010 Málaga, Spain; 3 Unidad de Gestión Clínica de Salud Mental, Instituto de Investigación Biomédica de Málaga (IBIMA), Hospital Regional Universitario, 29010 Málaga, Spain; 4 Departamento de Anatomía Humana, Medicina Legal e Historia de la Ciencia, IBIMA, Universidad de Málaga, 29071 Málaga, Spain; juan.suarez@ibima.eu; 5 Unidad de Parkinson y movimientos anormales, Servicio de Neurología, Hospital Clínic, 08036 Barcelona, Spain; etolosa@clinic.cat; 6 Centro de Investigación Biomédica en Red Sobre Enfermedades Neurodegenerativas (CIBERNED), Instituto de Salud Carlos III, 28029 Madrid, Spain; 7 Institut d’Investigacions Biomèdiques August Pi i Sunyer (IDIBAPS), Universitat de Barcelona, 08036 Barcelona, Spain; 8 Servicio de Neurología, Hospital Universitari Germans Trias i Pujol, 08916 Badalona, Spain; dvilas.germanstrias@gencat.cat; 9 Centro de Diagnóstico Biomédico, Departamento de Patología, Hospital Clinic de Barcelona, Universitat de Barcelona, 08036 Barcelona, Spain; ialdecoa@clinic.cat; 10 Banco de Tejidos Neurológicos del Biobanco, Institut d’Investigacions Biomèdiques August Pi i Sunyer (IDIBAPS), 08036 Barcelona, Spain; 11 Servicio de Radiología, Hospital Clínic, 08036 Barcelona, Spain; jberen@clinic.cat; 12 Servicio de Neurología, Hospital Universitario de Valme, 41014 Sevilla, Spain; damas.hermoso@gmail.com

**Keywords:** parkinson, α-synuclein, saliva, nitration, nitrative stress, lewy-type

## Abstract

*Background.* Salivary α-synuclein (aSyn) and its nitrated form, or 3-nitrotyrosine-α-synuclein (3-NT-αSyn), hold promise as biomarkers for idiopathic Parkinson’s disease (IPD). Nitrative stress that is characterized by an excess of 3-nitrotyrosine proteins (3-NT-proteins) has been proposed as a pathogenic mechanism in IPD. The objective is to study the pathological role of native αSyn, 3-NT-αSyn, and 3-NT-proteins in the saliva and submandibulary glands of patients with IPD. *Methods.* The salivary and serum αSyn and 3-NT-proteins concentration is evaluated with ELISA in patients and controls. Correlations of αSyn and 3-NT-proteins content with clinical features of the disease are examined. Immunohistochemical 3-NT-αSyn expression in submandibulary gland sections is analyzed. *Results.* (a) Salivary concentration and saliva/serum ratios of native αSyn and 3-NT-proteins are similar in patients and controls; (b) salivary αSyn and 3-NT-proteins do not correlate with any clinical feature; and (c) three patterns of 3-NT-αSyn-positive inclusions are observed on histological sections: rounded “Lewy-type” aggregates of 10–25 µm in diameter, coarse deposits with varied morphology, and spheroid inclusions or bodies of 3–5 µm in diameter. “Lewy-type” and coarse inclusions are observed in the interlobular connective tissue of the gland, and small-sized bodies are located within the cytoplasm of duct cells. “Lewy-type” inclusions are only observed in patients, and the remaining patterns of inclusions are observed in both the patients and controls. *Conclusions.* The patients’ saliva presents a similar concentration of native αSyn and 3-nitrotyrosine-proteins than that of the controls, and no correlations with clinical features are found. These findings preclude the utility of native αSyn in the saliva as a biomarker, and they indicate the absence of nitrative stress in the saliva and serum of patients. As regards nitrated αSyn, “Lewy-type” inclusions expressing 3-NT-αSyn are observed in the patients, not the controls—a novel finding that suggests that a biopsy of the submandibulary gland, if proven safe, could be a useful technique for diagnosing IPD. Finally, to our knowledge, this is also the first description of 3-NT-αSyn-immunoreactive intracytoplasmic bodies in cells that are located outside the nervous system. These intracytoplasmic bodies are present in duct cells of submandibulary gland sections from all subjects regardless of their pathology, and they can represent an aging or involutional change. Further immunostaining studies with different antibodies and larger samples are needed to validate the data.

## 1. Introduction

Human saliva contains α-synuclein (αSyn), a key protein in the pathogenesis of Parkinson’s disease (PD), and is an easily accessible fluid to be collected from patients with Parkinson’s disease [1,2,3,4,5,6,7,8]. Therefore, salivary αSyn holds promise as a biomarker for PD [6,7,8], although studies on native αSyn content in human saliva and its role in clinical features of PD have yielded conflicting results [1,2,3,4,5,6,7,8].

Lewy inclusions, neuropathological hallmarks of PD, are noted in salivary glands [9,10,11,12,13,14,15,16,17,18]. Lewy aggregates, as studied in the brain and the autonomic nervous system, express “physiological” or native αSyn as well as oxidized forms of the molecule, such as oligomeric, phosphorylated, and nitrated αSyn [9,10,11,12,13,14,15,16,17,18,19,20,21,22,23,24]. Oligomeric and phosphorylated αSyn expression and Lewy pathology have been widely explored in the salivary glands, but 3-nitrotyrosine-α-synuclein (3-NT-αSyn), the nitrated form of the protein, has not been analyzed [12,13,14,16,21]. Nitrated αSyn holds promise as a biomarker for PD because it is a component of proteinaceous aggregates or Lewy inclusions [9,10]. Excess amount of nitrated αSyn is recognized as a salient feature of α-synucleinopathies [9,10,19,24,25] and is thought to be neurotoxic and accelerate αSyn aggregation [17,25]. Excess amount of nitrated αSyn is associated with nitrative stress, a type of oxidative stress that is characterized by nitrative modifications of proteins and other molecules due to an excess of nitric oxide and reactive nitrogen species. Tyrosine residues of proteins such as αSyn are nitrated on the C-3 of the phenolic ring, giving rise to 3-nitrotyrosine proteins (3-NT-proteins) [10,19].

It is important to understand the role of nitrative stress and αSyn modifications in the saliva and salivary glands in PD pathogenesis. The objectives are as follows: (a) to study the expression of native and nitrated αSyn in the saliva and submandibulary gland in patients with idiopathic PD (IPD) and control subjects, (b) to detect the presence of nitrative stress in the saliva of patients and controls through the quantification of 3-NT-proteins, and (c) to explore the relationship of salivary αSyn and 3-NT-proteins with specific motor and non-motor features of the disease. Among salivary glands, the human submandibulary gland is the most active, producing ~65% of the total salivary volume [26].

## 2. Materials and Methods

### 2.1. Participants

For this cross-sectional and observational study, 50 patients with idiopathic PD and 30 control participants were enrolled at Hospital Valme, Sevilla, Spain. Five saliva samples were discarded due to high hemoglobin concentration or technical problems, and hence the final number of patients´ samples was 45. Patients were diagnosed with PD if they presented all three classic motor signs of Parkinsonism (bradykinesia, rigidity, and resting tremor) [7,27], as well as a reliable loss of dopamine-transporter signal on basal ganglia, as measured with ^123^I-Ioflupane DAT-SPECT [28]. All SPECT scans were performed, quantitatively analyzed, and visually assessed by expert physicians at the Service of Nuclear Medicine, following established criteria [28,29]. All patients had a disease duration longer than 3 years, and the age at PD onset was from 45 to 75 years. To exclude hereditary forms of Parkinsonism, those patients with atypical deficits, family members with PD, or younger than 45 years old were discarded. Control participants were recruited from volunteers, and they were group-matched by age and sex to PD subjects. Controls were excluded if they had a first-degree family member with PD or a neurological disorder.

### 2.2. Clinical Information

Standard demographic information was obtained from patients. Clinical data included the International Parkinson and Movement Disorder Society-sponsored revision of the Unified Parkinson’s Disease Rating Scale (MDS-UPDRS), the modified Hoehn–Yahr staging, and the modified Schwab–England activities of daily living scale. Age at PD onset and disease duration in years were also evaluated according to the year of motor symptoms onset, as reported by the patient. Individuals presenting with any liver, renal, cardiovascular, and hematological dysfunctions, as well as cancer, autoimmune disorders, or AIDS were not included because markers of oxidative and nitrative stress could be affected [30,31]. In addition, all participants were non-alcohol drinkers, non-smokers, and non-coffee drinkers [31,32,33,34]. Alcohol abuse was defined as drinking >210 g of alcohol per week. Smoking was defined as current smokers who consume cigarettes on a daily basis, or occasional smokers who consume cigarettes less than on a daily basis. Coffee drinking was defined as a person who intakes coffee drinks containing more than 300 mg of caffeine on a daily basis (e.g., more than 3 standard 8 oz cups of brewed coffee) [33].

Regarding medication, patients were treated with levodopa, dopamine agonists, and supportive medication that enhance dopaminergic effect. The antiparkinsonian medication was expressed as a levodopa equivalent dose (LED, mg per day), by using the following formula: LED = immediate-release levodopa × 1 + controlled-release levodopa × 0.75 + levodopa with entacapone × 1.33 + pramipexole × 100 + ropinirole × 20 + rotigotine × 30 + apomorphine × 10 + amantadine × 1 + rasagiline × 100 [35,36,37].

### 2.3. Serum and Saliva Collection and ELISA Analysis

Blood was collected by cephalic vein puncture. A total of 5 mL of blood was collected in gel-coated tubes to induce blood coagulation and to obtain serum (BD Vacutainer, Madrid, Spain). The blood serum was centrifuged at 2500 rpm for 10 min to separate clots and trapped cells, and then serum was immediately frozen at −80 °C in 0.5 mL aliquots. Three milliliters of saliva were collected in 5 mL polypropylene tubes (Eurotube DeltaLab, Barcelona, Spain). The saliva was centrifuged at 2500 rpm for 10 min to precipitate cells, and then the liquid portion was immediately frozen at −80 °C in 0.5 mL aliquots. Hemoglobin concentration in a fresh 0.5 mL saliva aliquot was quantified as recommended [7], and those samples with a hemoglobin concentration higher than 1200 mg/mL were discarded. The serum and saliva aliquots were unfrozen and sonicated with homogenizing solution (150 mM NaCl, 50 mM HEPES, 1 mM phenylmethylsulfonil fluoride, 0.6 µm leupeptin, 1% Triton X-100, pH 7.4).

The α-Synuclein concentration was evaluated with a commercially available Enzyme-linked Immunosorbent Assay kit (Human aSyn ELISA Kit, cat. #E09S0131, Shanghai BlueGene Biotech CO., LTD, Shanghai, China), following manufacturer’s instructions. Nitrative stress was evaluated through the quantification of the 3-NT-proteins concentration, by using a commercially available Enzyme-linked Immunosorbent Assay kit (Oxiselect Nitrotyrosine kit, Cell Biolabs Inc., catalog number STA-305-T, San Diego, CA, USA), following manufacturer’s instructions. Each sample was analyzed in duplicate (serum, 1/100 dilution; saliva, ½ dilution).

### 2.4. Immunohistochemical Study of Submandibulary Glands

Histological slides containing 5 µm sections of human submandibulary gland tissue were obtained from the IDIBAPS Biobank (Institut d’Investigacions Biomèdiques August Pi i Sunyer, University of Barcelona). Submandibulary gland tissue had been obtained through transcutaneous core needle biopsy with ultrasound guidance in patients with Parkinson’s disease (n = 6) and healthy controls (n = 6), as explained elsewhere [16]. Histological sections were deparaffinized and then stained against 3-nitrotyrosine α-synuclein (3-NT-αSyn) alone, or in combination with Iodotyrosine deiodinase (IYD). This latter enzyme was chosen because it is selectively expressed by excretory duct cells, not by secretory acinar cells (Fernández-Espejo, personal observation; see Figure 3). The antibodies used were as follows: alpha-synuclein monoclonal antibody (ThermoFischer Scientific, Waltham, MA, USA, Invitrogen, Carlsbad, CA, USA, Syn211, cat. #32-1800); anti-nitro-α/β-synuclein antibody (Merck, clone nSyn12, cat. 36-011); and IYD polyclonal antibody (ThermoFisher Scientific, cat. #PA5-63757). These antibodies are usually used in our laboratory. Sections were incubated in the primary antibodies and diluted 1/100 for 24 h at 4 °C. The next day the sections were incubated in the respective secondary antibody for 90 min: biotinylated goat anti-mouse IgG (1:500; cat. no. B7264, Sigma, St. Louis, MO, USA) or biotinylated donkey anti-rabbit IgG (1:500; cat. no. RPN1004, Amersham, Little Chalfont, UK). The sections were then incubated in ExtrAvidin peroxidase (Sigma) diluted 1:2000, in darkness at room temperature for 1 h. Finally, immunoreactivity was revealed with 0.05% diaminobenzidine (DAB; Sigma) diluted in 0.1 M phosphate-buffered saline (PBS) or DAB and 0.05% nickel ammonium sulfate diluted in PBS. The peroxidase reaction was activated after the addition of 0.03% H_2_O_2_.

### 2.5. Histological Examination

All sections were reviewed by researchers blinded to the clinical information (E.F.E. and J.S.). Sections with positive immunoreactivity were visualized using a standard optical microscope (Nikon Instruments Europe B.V., Amstelveen, the Netherlands), coupled to the NIS-Elements Imaging Software 3.00 (Nikon). We screened 4–6 serial sections per subject with the anti-nitro-α/β-synuclein antibody and the anti-IYD polyclonal antibody. The degree of immunoreactive inclusions within different regions of the submandibulary gland was assessed in contiguous tissue sections according to a five-point rating scale: not detectable (0), mild (1), moderate (2), frequent (3), and very frequent (4). These regions encompassed acini, ducts, and interlobular connective tissue (that contain blood vessels and autonomic nerves supplying the gland). The presence of immunoreactive inclusions was also assessed in other intraglandular cells such as adipocytes, endothelial, and mononuclear blood cells [24].

### 2.6. Statistics and Ethics

Comparisons of dichotomous variables were carried out with the χ^2^ test. As for quantitative variables, two groups were compared with Student’s t test, or the Mann-Whitney U test if distribution was a non-parametric one. Correlations between two dependent variables were carried out with the Pearson’s test, or the Spearman’s test in non-parametric distribution. Normalization of data was verified with the Shapiro-Wilk test. All the protocols were approved by the Internal Ethics and Scientific Board of Hospital Universitario Valme (ref. 10/05/2018), University of Seville (CEI27/05/2010), and Research Ethics Committee of Junta de Andalucia (PEIBA; CEI Sevilla Sur, ref. 2017121418738). The subjects’ consent was obtained according to the Declaration of Helsinki (BMJ 1991; 302: 1194).

## 3. Results

### 3.1. Participant Characteristics

Basic demographic features are not found to be different between patients and controls, as shown in Table 1. Clinical parameters of patients are also shown in Table 1.

### 3.2. Salivary and Serum Native αSyn

**Figure 1:**
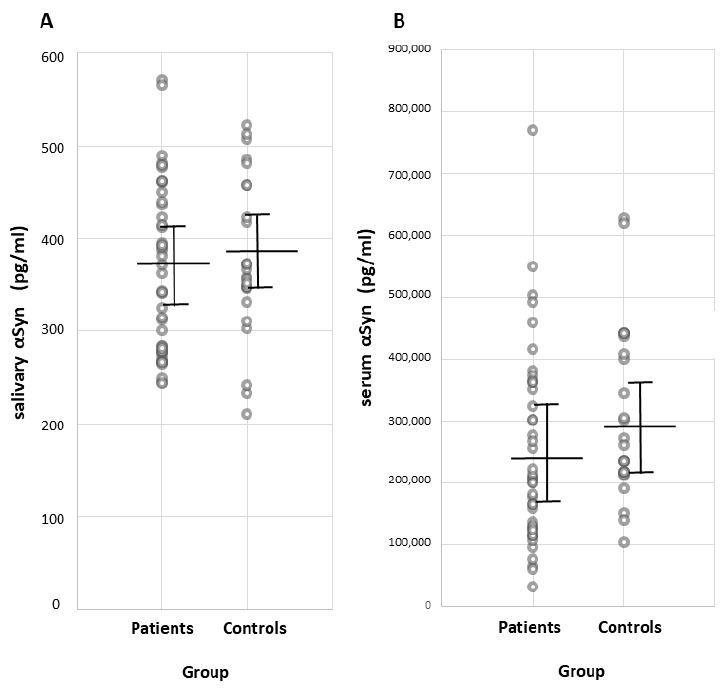
Individual total αSyn concentration (pg/mL) in (**A**) saliva and (**B**) serum in patients with IPD and control participants, as measured with ELISA. Mean and standard deviation are represented with solid lines. Abbrev.: αSyn, α-synuclein; IPD, idiopathic Parkinson’s disease; ELISA, enzyme-linked immunosorbent assay.

Total αSyn concentration in the saliva and serum, as measured through ELISA, does not differ between patients and controls (Table 1). Individual αSyn concentration values are shown in Figure 1. The saliva/serum ratio of native αSyn is similar in both patients and controls (~1:700, Table 1). No correlations are found between the native αSyn concentration in saliva and demographic and clinical parameters, as shown in Table 2.

### 3.3. Salivary and Serum 3-Nitrotyrosine Proteins, and Nitrative Stress

**Figure 2:**
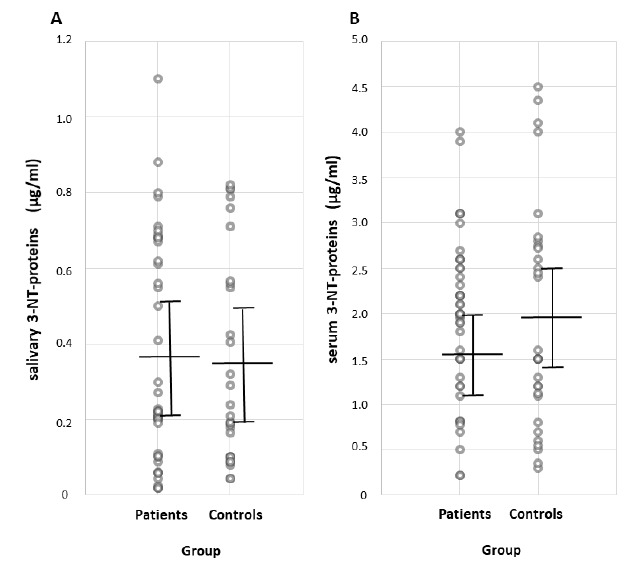
Individual 3-NT-proteins concentration (µg/mL) in (**A**) saliva and (**B**) serum in patients with IPD and control participants, as measured with ELISA. Mean and standard deviation are represented with solid lines. Abbrev.: 3-NT-proteins, 3-nitrotyrosine proteins; IPD, idiopathic Parkinson’s disease; ELISA, enzyme-linked immunosorbent assay.

The concentration of 3-NT-proteins is measured in saliva and serum, since it is a biochemical parameter that could be indicative of nitrative stress. The total 3-NT-proteins concentration in saliva and serum, as measured through ELISA, does not differ between patients and controls (Table 1). Individual 3-NT-proteins concentration values are shown in Figure 2. The saliva/serum ratio of 3-nitrotyrosine proteins is not found to be different in patients relative to controls (Table 1). No significant correlations are found between the 3-NT-proteins concentration in saliva and demographic and clinical parameters, as shown in Table 2.

### 3.4. 3-Nitrotyrosine-αSyn in the Submandibulary Gland

**Figure 3:**
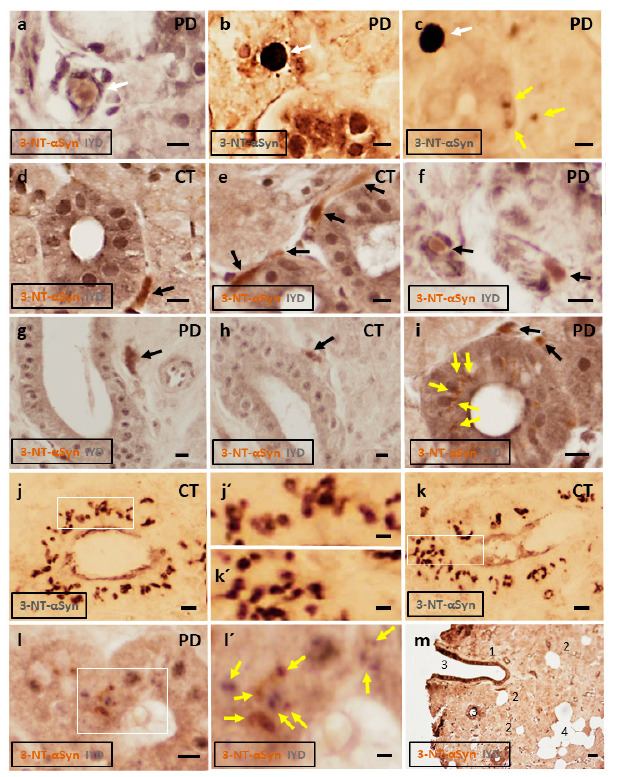
Representative photomicrographs of submandibulary gland sections in patients with idiopathic Parkinson’s disease and controls, after immunostaining against 3-NT-αSyn (brown color) and IYD (grey color), or against 3-NT-αSyn alone (dark grey). (**a**–**c**) Rounded “Lewy-type” inclusions of 10–25 µm in diameter are observed in the interlobular connective tissue (white arrows). These inclusions are surrounded by a halo (**a**) or radiating fibrils (**b**,**c**). “Lewy-type” inclusions are observed in patients, not in controls. (**d**–**i**) 3-NT-αSyn-positive coarse inclusions are observed within the interlobular tissue, many of them close to duct cells (black arrows). Ribbon-like, spindle-like, and ovoid deposits are seen. (**c**,**i**–**l**) 3-NT-αSyn-immunoreactive spheroid bodies of 3–5 µm in diameter are observed in the cytoplasm of duct cells (yellow arrows). High-magnification images of intracytoplasmic bodies are seen in (**j´**,**k´**,**l´**). Bodies are observed in the patients and controls. (**m**) Low-magnification and double-stained image of a submandibulary gland section showing main cell types (1, interlobular connective tissue; 2, acini with IYD-negative cells; 3, ducts with IYD-positive cells; 4, adipocytes). Abbrev.: 3-NT-αSyn, 3-nitrotyrosine α-synuclein; IYD, iodotyrosine deiodinase; PD, Parkinson´s disease; CT, control gland. Bars: 10 µm in (**a**–**l**); 5 µm in (**j´**,**k´**,**l´**); 50 µm in m.

Immunohistochemical study of the submandibulary gland reveals three patterns of inclusions expressing 3-NT-αSyn: (1) rounded “Lewy-type” aggregates, (2) coarse aggregates of varied morphology, and (3) small-sized bodies of spheroid shape (Figure 3). First, rounded “Lewy-type” inclusions are located within the interlobular connective tissue (Figure 3a–c). They resemble Lewy bodies because they are 10–25 µm in diameter, and show a peripheral halo or radiating fibrils, as described elsewhere [9,38,39]. Importantly, these “Lewy-type” inclusions are observed in patients with IPD, not in controls. Second, the interlobular connective tissue also contains coarse inclusions of varied morphology, including ribbon-like, spindle-like, and ovoid shapes (Figure 3d–i). It seems that the different shapes might result from a different viewing angle. Coarse inclusions are found in 5/6 IPD patients and 4/6 controls. Third, rounded bodies with a diameter of 3–5 µm are seen within the cytoplasm of IYD-positive duct cells (Figure 3c,i–l). All sections from the patients and control subjects showed 3-NT-αSyn-positive bodies. IYD-negative acinar cells, adipocytes, endothelial or blood cells are devoid of immunoreactive inclusions. A role of β-synuclein in immunoreaction signals cannot be discarded, since an anti-α/βsynuclein antibody was used. Density of the different patterns of inclusions and their location in the submandibulary glands in patients and controls are shown in Table 3. Finally, there were no significant differences in age (patients with IPD, 65.3 ± 8 years; control participants, 61.8 ± 10 years), and gender between the patients and controls.

## 4. Discussion

In this cross-sectional study, the concentration of native αSyn in the saliva is found to be similar in patients with IPD and control participants, which is consistent with many other studies [1,2,3,6,7,8]. No correlations are detected between the native αSyn concentration and motor and non-motor features of IPD, in accordance with most studies [1,5,7,8]. Blood serum contains αSyn, without difference in protein levels between patients and controls [6,7]. The results of the present study would preclude using native αSyn in the saliva as a biomarker for IPD. Regarding 3-nitrotyrosine proteins, these molecules are quantified in saliva and serum to detect the presence of nitrative stress, a type of oxidative stress that is characterized by elevated levels of 3-NT-proteins. No differences are observed between patients with IPD and controls, indicating the absence of nitrative stress in the saliva or serum of IPD patients.

The saliva/serum ratio of native αSyn is ~1:700 in both patients and controls, a result that indicates a normal or “physiological” secretion of native α-synuclein in forming saliva in patients with PD. This result is of interest because the source of salivary native αSyn is unknown. Given that αSyn can be secreted by neurons [40], it is possible the salivary nerves release αSyn into saliva. However, it is also possible that αSyn derives from the cellular secretion of saliva or blood. Our findings support the hypothesis that salivary native αSyn comes from blood, because the saliva/serum ratio was similar in all subjects regardless of the pathology. The saliva/serum ratio of nitrated proteins is similar in patients and controls, a finding that confirms the absence of nitrative stress in these biofluids in patients with IPD.

As regards the histological study, the submandibulary gland is an exocrine gland with multiple lobules that contain secretory acinar cells. These lobules are separated by connective tissue that contain excretory ducts, blood vessels, and autonomic nerves supplying the gland. The immunohistochemical study reveals three different patterns of 3-NT-αSyn immunoreactivity, including rounded “Lewy-type” aggregates, coarse inclusions of varied morphology, and small-sized intracytoplasmic bodies.

Rounded “Lewy-type” inclusions are seen in the interlobular connective tissue, and they are considered as specific to PD because they are observed in patients, not in controls. These inclusions resemble Lewy bodies because they are 10–25 µm in diameter, and they show a peripheral halo or radiating fibrils [9,19,38,39]. The detection of “Lewy-type” aggregates expressing nitrated αSyn in the submandibulary glands of patients is, to the best of our knowledge, a novel finding. All previous studies on Lewy pathology in human submandibulary glands are based on oligomeric and phosphorylated αSyn, other oxidation-related forms of the molecule [13,14]. This is an important issue because formation of Lewy aggregates is linked to the etiology of Parkinson’s disease and other neurodegenerative disorders [9,41].

The interlobular connective tissue also contains coarse inclusions of varied morphology. They are observed in histological sections from patients and controls. These deposits have ribbon-like, spindle-like, and ovoid shapes; morphologies that are the likely consequence of different viewing angles. Of note is that these morphologies resemble previous descriptions of phosphorylated-αSyn-positive inclusions within the interlobular connective tissue of salivary glands [14,16,24,42,43,44]. Therefore, these morphological shapes seem to be a common feature of αSyn-positive inclusions within the interlobular tissue of salivary glands.

Previous double-staining studies with antibodies against neural markers such as neurofilaments or protein-G product indicate the neuronal identity of “Lewy-type” and coarse deposits within the interlobular tissue [42,43,44]. Although we are unable to confirm the neuronal identity of these inclusions, their location within the interlobular connective tissue (where salivary neural fibers supplying the submandibulary gland are located) would suggest that these 3-NT-αSyn-positive aggregates derive from neuronal secretion [40]. However, it cannot be disregarded that aggregated nitrated αSyn can derive from cells other than neurons, and more studies are required.

Another novel finding is that the cytoplasmic staining of IYD-positive duct cells is condensed into defined small-sized spheroid inclusions or bodies that express nitrated αSyn. Intracytoplasmic inclusions of nitrated αSyn are well documented in the brain [45,46,47] but, to our knowledge, this is the first description of 3-NT-αSyn-immunoreactive bodies within the cytoplasm of non-neural cells, specifically within duct cells of the submandibulary gland. These bodies have a diameter of 3–5 µm, and they are frequently observed in all subjects regardless of their pathology. These observations suggest that these small-sized inclusions are not of pathological significance, and it is possible that they represent an aging or involutional change in duct cells. Further studies are needed to analyze these intracytoplasmic bodies, and their physiological or pathological significance.

There are some limitations to our study. Thus, it is important to note that several authors have proposed that some immunoreactions in salivary glands may be explained as cross-reactions due to endogenous enzyme activity [24], and that patterns of α-synuclein-immunoreactive inclusions are highly dependent on the specificity of primary antibodies [24,42,43,44]. In addition, a role for nitro-β-synuclein in immunoreactions cannot be discarded because an anti-nitro-α/β-synuclein antibody was used. Further validation of our immunostaining findings with additional antibodies is needed to validate the usefulness of nitrated αSyn expression in the submandibulary gland as a diagnostic tool for PD. Finally, the sample size in the histological study is small, and the results must be confirmed by means of a larger sample in future studies.

## 5. Conclusions

The saliva of patients with idiopathic PD presents a similar concentration of native αSyn and 3-nitrotyrosine-proteins than that of controls, and no correlations with clinical features are found. These findings preclude the utility of native αSyn as a biomarker, and they indicate the absence of nitrative stress in the saliva and serum of patients. As regards nitrated αSyn, “Lewy-type” inclusions expressing 3-NT-αSyn are observed in patients, not in controls. It is a novel finding which suggests that a biopsy of the submandibulary gland, if proven safe, could be a useful technique for diagnosing IPD. It is also described for the first time the presence of small-sized bodies within the cytoplasm of cells that are located outside the nervous system. These intracytoplasmic bodies are present in duct cells of submandibulary gland sections from all subjects regardless of their pathology, and they can represent an aging or involutional change. Finally, it is important to further validate the immunostaining findings with additional antibodies and larger samples.

## Figures and Tables

**Table 1:** Demographic and clinical parameters, as well as concentration of salivary and serum αSyn and 3-nitrotyrosine proteins, in patients with IPD and control subjects.

	IPD (n = 45)	Control (n = 30)	P
**Demographic and clinical parameters**			
Age (years)	61.4 ± 18.5	59.6 ± 11	NS
Gender, male n (%)	27(60)	12 (40)	NS
Body mass index	23.1 ± 2	24.8 ± 2.5	NS
Education (years)	17.8 ± 2.1	17.3 ± 2.2	NS
Levodopa equivalent dose (mg per day)	595.9 ± 650		
Disease duration (years)	9.9 ± 6.8		
Age at IPD onset (years)	57.9 ± 13.1		
Hoehn-Yahr stage	2.1 ± 0.8		
Modified Schwab-England	86 ± 25		
MDS-UPDRS part III (on)	24 ± 12		
Total MDS-UPDRS (I-III) (on)	37.2 ± 20		
MDS-UPDRS part IV	1.2 ± 2.4		
**Native αSyn concentration (pg/mL)**			
Saliva	361.9 ± 89	372.1 ± 91	NS
Serum	244,789 ± 114,017	297,783 ± 110,992	NS
Saliva/serum αSyn ratio	0.0015 ± 0.001	0.0013 ± 0.001	NS
**3-Nitrotyrosine proteins concentration (µg/mL)**			
Saliva	0.35 ± 0.28	0.33 ± 0.27	NS
Serum	1.52 ± 0.87	1.94 ± 1.23	NS
Saliva/serum 3-NT-proteins ratio	0.27 ± 0.25	0.21 ± 0.15	NS

Mean ± SD. Statistical comparisons were carried out with the χ^2^ test (dichotomous variables) or the Student’s t-test (quantitative variables). Abbrev.: IPD, idiopathic Parkinson’s disease; NS, nonsignificant; UPDRS, Unified Parkinson’s Disease Rating Scale; P, probability; αSyn, α-synuclein; 3-NT-proteins, 3-nitrotyrosine proteins.

**Table 2:** Correlation of native αSyn and 3-nitrotyrosine proteins concentration in the saliva with demographic and clinical parameters in patients with idiopathic Parkinson’s disease.

Parameters	Native αSyn	3-Nitrotyrosine Proteins
Age (years)	0.192	0.027
Body mass index	0.215	−0.058
Education (years)	0.071	−0.065
Levodopa equivalent dose (mg per day)	0.166	−0.209
Disease duration (years)	0.044	−0.098
Age at IPD onset (years)	−0.039	0.014
Hoehn-Yahr stage	0.179	0.006
Modified Schwab-England	−0.102	−0.161
MDS-UPDRS part III (on)	0.048	−0.107
Total MDS-UPDRS (I-III) (on)	0.039	−0.125
MDS-UPDRS part IV	0.206	−0.198

Mean ± SD. Statistical correlation was carried out with the Pearson´s test. No significant correlations were found. Abbrev.: IPD, idiopathic Parkinson’s disease; αSyn, α-synuclein; NS, no significant; MDS-UPDRS, International Parkinson and Movement Disorder Society-sponsored revision of the Unified Parkinson’s Disease Rating Scale.

**Table 3:** Demographic, clinical and neuropathological data from all cases studied with idiopathic PD and control participants.

Demographic and Clinical Parameters					Patterns of Inclusions (Location)			
Case	Gender	Age (y)	H-Y	Disease Duration (y)	“Lewy-Type” Inclusions (Interlobular Connective Tissue)	Coarse Aggregates (Interlobular Connective Tissue)	Small-Sized Bodies (Cytoplasm of Duct Cells)	Other Inclusions (Acinar Cells, Adipocytes, Endothelial or Blood Cells)
PD1	F	76	2	6	1	2	4	0
PD2	M	73	2.5	8	2	2	3	0
PD3	M	56	2	6	2	2	3	0
PD4	M	55	2	7	2	2	4	0
PD5	M	70	1.5	5	1	0	4	0
PD6	F	71	2.5	7	2	1	3	0
CT1	M	66			0	2	4	0
CT2	F	55			0	2	3	0
CT3	F	47			0	0	4	0
CT4	F	66			0	2	4	0
CT5	M	77			0	2	3	0
CT6	M	60			0	0	3	0

The degree of immunoreactive inclusions within different regions of the submandibulary glands was assessed according to a five-point rating scale: not detectable (0), mild (1), moderate (2), frequent (3), and very frequent (4). Abbrev.: PD, Parkinson’s disease; CT, control; F, female; M, male; H-Y, Hoehn–Yahr stage.

## Data Availability

The data presented in this study are available on request from the corresponding authors.
